# Colonic Manometry in Pediatric Patients with Spina Bifida: Results from a Retrospective Cohort Study

**DOI:** 10.3390/children12020184

**Published:** 2025-02-04

**Authors:** Albert Yuh Chyuan Shan, Barry Duel, Timothy Phillips, Paula Buchanan, Leonel Rodriguez, Dhiren Patel

**Affiliations:** 1 Division of Pediatric Gastroenterology, Hepatology and Nutrition, Cardinal Glennon Children’s Medical Center, Saint Louis University School of Medicine, St. Louis, MO 63104, USA; dhiren.patel@slucare.ssmhealth.com; 2 Division of Pediatric Urology, Cardinal Glennon Children’s Medical Center, Saint Louis University School of Medicine, St. Louis, MO 63104, USA; barry.duel@slucare.ssmhealth.com (B.D.); timothy.phillips@slucare.ssmhealth.com (T.P.); 3 Advanced HEAlth Data (AHEAD) Institute, Saint Louis University School of Medicine, St. Louis, MO 63104, USA; paula.buchanan@health.slu.edu; 4 Section of Pediatric Gastroenterology, Hepatology and Nutrition, Department of Pediatrics, Yale University School of Medicine, New Haven, CT 63104, USA; leonel.rodriguez@yale.edu

**Keywords:** neurogenic bowel, anorectal manometry, colonic transit time, antegrade continence enema, colonic motility, spina bifida

## Abstract

Background/Objectives: Patients with spina bifida (SB) commonly experience neurogenic bowel dysfunction, characterized by defecation-related symptoms. While anorectal dysfunction and slow transit constipation (STC) have been implicated, the role of colonic motility in SB remains unclear. This study aimed to evaluate colonic motility in SB patients with refractory bowel dysfunction. Methods: This retrospective cohort study included SB patients who failed the repeated optimization of a bowel regimen including stimulant laxatives and subsequently underwent anorectal manometry (ARM), colonic transit time (CTT) studies, or colonic manometry (CM). Diagnostic findings were analyzed alongside treatment outcomes. Results: A total of 13 patients with myelomeningocele were included; one declined further treatment, and 12 underwent treatment optimization, with four achieving bowel continence. Of the five patients who proceeded with advanced motility testing, two had abnormal ARM findings, one of three had abnormal CTT results, and all five had normal CM findings. Conclusions: These findings suggest that anorectal dysfunction or STC may play a larger role in refractory bowel symptoms, while colonic motility appears to be preserved, and this highlights the importance of maximizing conservative therapies, particularly with stimulant laxatives, before pursuing invasive tests or surgical interventions for bowel dysfunction in this population.

## 1. Introduction

Spina bifida (SB) is a congenital malformation of the spinal cord that affects multiple organ systems and can lead to significant long-term disabilities, even after surgical repair. It occurs due to the failure of neural tube closure during the third to fourth week of gestation, resulting in open neural tube defects [1]. Depending on the level of involvement, SB is associated with various functional impairments, including cognitive deficits, paralysis and sensory loss in the lower limbs, and abnormal bladder and bowel function. Constipation and/or fecal incontinence affect up to 86% of patients with SB, likely due to neurogenic bowel dysfunction involving sensory and motor disturbances, which significantly impacts quality of life [2].

Our understanding of the effect of SB on gastrointestinal motility physiology remains limited. Some studies have reported abnormalities in colonic transit and anal sphincter function in patients with SB, while others have noted anal spasms and prolonged sphincter relaxation during rectal balloon distention in patients with other spinal lesions (e.g., tethered cord, fatty filum, and spinal lipoma) [2,3,4,5]. To our knowledge, there are currently no data on colonic motility in patients with SB. Here, we present our experience with colonic motility assessment in SB patients experiencing refractory bowel dysfunction.

## 2. Materials and Methods

A retrospective cohort study was conducted at a single institution from September 2019 to June 2022, involving patients with SB and bowel dysfunction refractory to medical therapy. Institutional review board approval was obtained. Patients between 4 and 18 years old with SB and constipation and/or fecal incontinence were included, while those with anorectal malformations, Hirschsprung disease, sacral agenesis, previous spinal injury due to trauma or tumor, or overt secondary causes were excluded.

Data collection included demographics, the lesion level, the SB subtype, mobility, medical and surgical history, key physical exam findings, labs, and previous medical treatment. SB lesion levels were categorized into four groups based on nerve innervation: (1) T8 and above, (2) T9–L2, (3) L3–S1, and (4) S2 or lower. Mobility was classified as ambulatory, semi-ambulatory, or wheelchair-bound. The symptoms of bowel dysfunction prior to treatment optimization were categorized as constipation, fecal incontinence, or both.

Referred patients were encouraged to complete a treatment optimization and evaluation process following the facility’s pathway, as illustrated in Supplemental Figures S1 and S2. Medically refractory bowel dysfunction was defined as the failure to respond to optimized conventional therapy (routine use of maximum doses of oral or enteral stimulants combined with one or more other pharmacologic therapies) for at least eight weeks. Those who failed to respond underwent more advanced testing, including colonic transit time (CTT), anorectal manometry (ARM), and/or colonic manometry (CM). Data on surgical procedures performed following testing, if applicable, were also collected.

### 2.1. Gastrointestinal Motility Studies

#### 2.1.1. Colon Transit Time (CTT)

The simplified method was used to assess colonic transit, utilizing a gelatin capsule containing 24 radio-opaque single-pattern markers. Patients with fecal impaction underwent a bowel cleanout prior to capsule ingestion. Patients were instructed to discontinue stimulant laxatives or anti-diarrheal agents 72 h before the study and to avoid all laxatives, enemas, or suppositories for five days following capsule ingestion. On day 0, the capsule was taken orally, and an abdominal X-ray was performed on day 5. An abnormal CTT was defined as the retention of ≥6 markers [6,7,8].

#### 2.1.2. Anorectal Manometry (ARM)

Preparation included an age-dependent single dose of a glycerin or bisacodyl suppository three hours before the study. Studies were performed with a high-resolution solid-state catheter, following previously published protocols [9]. The variables recorded included the anal resting pressure (mmHg), squeeze pressure (mmHg), and presence and quality of the rectal–anal inhibitory reflex (RAIR), defined as present when a drop of ≥15% in the resting pressure was observed; prolonged RAIR and anal spasms during sustained rectal balloon inflation were also assessed. Normative data published by Kumar et al. were used for comparison [9,10].

#### 2.1.3. Colon Manometry (CM)

Patients underwent bowel preparation the day before the procedure. The procedure was performed as per previously published guidelines [9]. Key parameters recorded included the gastric–colonic response to a meal (GCS) and the presence of high-amplitude propagating contractions (HAPCs). A normal CM study was defined as the presence of both GCS and HAPCs [9].

- GCS: Visual observation of increased post-prandial colonic activity after meal challenge compared to fasting recording.
- HAPCs: Contractions lasting ≥10 s and propagating more than 30 cm with an amplitude of ≥60 mmHg, defined as normal when migrating from the cecum and reaching the recto-sigmoid junction.

### 2.2. Statistical Analysis

We used descriptive statistics to summarize the basic demographic and clinical characteristics of the study population. We summarize continuous variables using medians and interquartile ranges (IQR) and categorical variables as proportions.

## 3. Results

A total of 13 patients were included, all with myelomeningocele, with a median (IQR) age of 7 (5–11) years old; nine (69%) were male and four (31%) were female. Three out of these 13 (23.1%) had lesions located at T9–L2, seven (53.8%) at L3–S1, one (7.7%) at S2 and below, and two (15.4%) had an unknown level of lesion. In terms of their bowel dysfunction type before optimization, eight (61.5%) of the patients presented with constipation, one (7.7%) had fecal incontinence, three (23%) had both, and information was not available in one (7.7%) patient. None of them were receiving the optimal enteral bowel regimen at the first visit; nine of them (69%) were not receiving any type of enteral bowel regimen.

**Figure 1:**
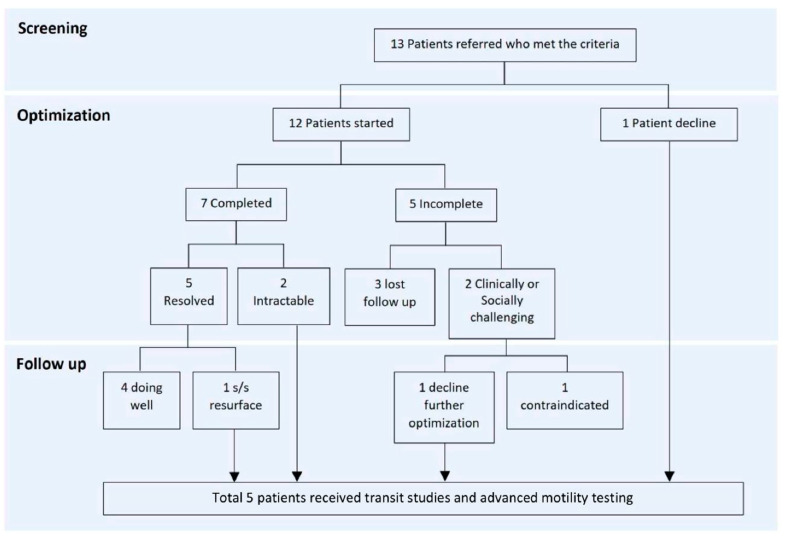
Treatment optimization process.

Figure 1 outlines the process for patients receiving therapy and undergoing motility studies. Of the 13 patients, one declined further treatment and the remaining 12 patients underwent treatment optimization; seven completed it, while five did not, with a total of four (33%) successful cases (all presented with constipation and remained well controlled until the end of the study). Among these four patients, two had lesions at T9–L2, one at S2 or lower, and one had an unknown lesion level. Of the three patients with refractory bowel dysfunction post-optimization, one had a lesion at T9–L2, and two had lesions at L3–S1. Ultimately, five patients who either failed medical interventions or declined optimization proceeded with more advanced testing.

Detailed demographic characteristics and results, for those with motility testing, are described in Table 1. In summary, two (40%) presented with constipation, one (20%) with incontinence, and two (40%) with both. All five patients underwent ARM and CM, and three (60%) patients received all testing (CTT, ARM, and CM).

For ARM, all patients had normal resting pressure and a positive RAIR response. One patient (patient 3) had prolonged anal sphincter relaxation during RAIR, and one out of the three had low squeeze pressure (patient 2). Of the three CTT tests, two of them were normal, and one was abnormal. All had normal CM results. Patient 3 underwent an antegrade continence enema (ACE) procedure.

## 4. Discussion

In our cohort of children with SB, all of whom had myelomeningocele, we observed normal CM findings in all patients, regardless of the bowel dysfunction type or the results of CTT and ARM. To our knowledge, this is the first early case series to describe CM findings in patients with SB and refractory bowel dysfunction.

Colonic motility is governed by the intricate interaction of neuromuscular components, with the Interstitial Cells of Cajal (ICC) playing a critical role in regulating motor patterns such HAPCs. The enteric nervous system, located entirely within the gastrointestinal tract, operates autonomously but is modulated by the extrinsic nervous system [11]. CM is the gold standard in assessing colonic neuromuscular function, although studies on colonic motility in children with spinal cord injury are scarce. Maartji et al. reported the predictive value of CM and contrast enema before cecostomy placement in children with defecation disorders, which included six patients with spinal abnormalities [12]. Among these, two exhibited HAPCs limited to the proximal colon, while one had the complete absence of HAPCs. Notably, the study did not specify the exact spinal abnormalities and used a catheter with a limited number of widely spaced sensors (eight recording sites spaced 10–15 cm apart), which may have reduced the sensitivity in detecting CM parameters compared to high-resolution manometry. Interestingly, a recent study on tethered cord syndrome (TCS) found that the CM results in children with TCS after detethering surgery were similar to those in a matched cohort with functional constipation. While both TCS and SB, specifically myelomeningocele, involve spinal cord injury, their pathophysiology differs. TCS leads to excessive traction, impairing spinal cord perfusion and metabolism, which can improve following detethering [13]. In contrast, myelomeningocele involves an initial failure of neural tube closure followed by ongoing prenatal injury, leading to progressive neurological deterioration. Our findings could suggest that, while the extrinsic nervous system is affected in SB, the intrinsic enteric nervous system could possibly be maintained in patients with myelomeningocele, resulting in normal CM findings. No colonic manometry results for other neurogenic bowel conditions are available in the literature. However, since the extrinsic nervous supply is typically affected while the intrinsic enteric nervous system remains intact regardless of the etiology, our findings may be generalizable to most neurogenic bowel conditions.

Contrary to reports in the literature suggesting that SB patients typically exhibit lower resting pressures on ARM, all patients in our study demonstrated normal resting pressures. Notably, in patient 3, we identified abnormal prolonged sphincter relaxation, aligning with prior studies that have reported significantly longer durations of the RAIR in children with myelomeningocele [3]. Patient 5 exhibited abnormal CTT, a frequent finding in SB. These tests assess different aspects of bowel function. CTT reflects the overall transit time and can be influenced by multiple factors. While colonic motility is one factor, other variables, such as extrinsic nervous regulation, the anorectal complex (continence and defecation regulation), sensory issues in SB, developmental maturity, central muscle tone, mobility, and behavior, also play significant roles. Given that SB patients often display normal CM results but present with abnormal ARM or CTT findings, these observations suggest that anorectal dysfunction or STC may play a more important role in defecation symptoms than colonic dysmotility in this population.

The evaluation and treatment approaches for SB patients are currently limited, with recent studies proposing stepwise approaches, including conservative therapy, oral laxatives, rectal therapy, trans-anal irrigation, and the ACE procedure [14]. Despite the advocacy for stimulant laxatives to improve colonic transit and facilitate recto-sigmoid emptying, only one out of twelve patients in our study was receiving stimulant laxative therapy before optimization. Interestingly, four patients who presented with constipation-type bowel dysfunction successfully achieved continence after optimizing their regimens with stimulant laxatives. The underutilization of stimulant laxatives may be explained by the scope of practice of primary providers in this patient population, where concerns about potential side effects and dependency on stimulants may have discouraged their use. However, a recent study on functional constipation demonstrated that bisacodyl is safe, well tolerated, and can be successfully discontinued in the majority without dependency in patients with functional constipation [15]. Increased education on the safety and long-term use of stimulant laxatives, especially in first-line providers, could enhance the treatment of bowel dysfunction in SB patients, potentially reducing the reliance on invasive testing and surgical interventions.

A prospective study by Daeze et al. demonstrated that patients with SB who have both normal CTT and normal anal resting pressure are likely to experience spontaneous fecal continence [5]. We observed a similar trend, as patient 1 responded well to the initial optimization, with recurrence suspected due to non-compliance. CM has shown promise in predicting and guiding surgical treatment for patients with refractory functional constipation, both initially and in assessing post-surgical improvements in colonic motility to guide decisions on ACE weaning and ostomy closure [16,17]. However, its utility in managing neurogenic bowels in children with SB is unclear. As CM often tends to be normal in these patients’ post-optimization, and none had ACE procedures based solely on the CM results, it may not influence decision-making as it does in functional constipation. Therefore, treatment decisions for SB, such as ACE or ostomy placement, are more likely driven by clinical factors aimed at improving quality of life due to refractoriness to medical treatment, rather than the CM results. This approach mirrors that used for functional constipation unresponsive to conventional therapy. It is possible that CM could become abnormal over time after prolonged treatment failure and progressive colonic distension, following similar clinical progression as refractory functional constipation. Additionally, patients with sacral-level lesions (L3–S1) may have a higher optimization failure rate compared to those with higher thoracic/lumbar lesions (T9–L2), suggesting a potential correlation between the lesion level and refractoriness. Future studies stratifying patients according to the level of lesions could provide further insights, refining the use of advanced motility testing and therapeutic interventions.

Our study has several limitations, including its retrospective design and small sample size. Only the severe form—myelomeningocele—was included, which we believe provides more insight, especially given the normal results; however, this may limit its generalizability. The universally established normal values for motility parameters in ARM and CM, particularly in children, are lacking and remain an ongoing concern among providers. However, efforts have been made to standardize the interpretation and define what is considered normal at present. The sustainability of the optimization outcome is limited to the duration of the study. Additionally, in congruence with any retrospective study design, we also had some missing data elements, limiting our ability to evaluate and interpret all data of interest. Despite these limitations, we hope that our initial report of colonic manometry findings in patients with SB and refractory bowel dysfunction provides a framework and encourages future large, multi-center, prospective cohort collaborations to enhance the generalizability and reliability.

## 5. Conclusions

Bowel dysfunction is nearly universal in patients with SB, with varying degrees in severity. While anorectal dysfunction and STC are frequently observed, our early findings suggest that colonic manometry evaluations are often normal. Optimizing conservative therapy, particularly the use of stimulants for severe constipation, is crucial before considering invasive tertiary motility evaluations like CM. However, since the CM results tend to be normal in these patients, treatment decisions are more likely guided by clinical judgment rather than test results.

## Figures and Tables

**Table 1:** Demographics and results of motility testing (N = 5).

Patient	1	2 *	3	4	5
Age	17	10	4	6	5
Gender	F	F	M	F	M
Level	L3–S1	L3–S1	L3–S1	L3–S1	T9–L2
Mobility	Walking	Walking	Semi-ambulate	Walking	Wheelchair
Morbidity	Tethered cord post-release	Hydrocephalus with VP ^7^ shunt Celiac disease	Hydrocephalus with VP shunt	n/a	Hydrocephalus post-ETV ^8^
Bowel dysfunction type	Constipation	Fecal incontinence	Both	Constipation	Both
CTT ^1^	Normal	n/a	Normal	n/a	Abnormal
ARM ^2^					
Average anal resting pressure (RP) (mmHg)	91	67	42	56	51
Squeeze pressure (SP)	229	50	n/a	131	n/a
RAIR ^3^	Present	Present	Present	Present	Present
Interpretation	Normal RP Normal SP RAIR present	Normal RP Low SP RAIR present	Normal RP RAIR present and prolonged	Normal RP Normal SP RAIR present	Normal RP RAIR present
CM ^4^					
Fasting HAPC ^5^	Absent	Absent	Present	Absent	Present
Post-prandial HAPC	Present	Present	Present	Present	Present
Post-bisacodyl HAPC	Present	Present	Present	Present	Present
Interpretation	Normal	Normal	Normal	Normal	Normal
Post-CM surgery	None	None	ACE ^6^	None	None

^1^ CTT: colonic transit time; ^2^ ARM: anorectal manometry; ^3^ RAIR: recto-anal inhibitory reflex; ^4^ CM: colonic manometry; ^5^ HAPC: high-amplitude propagating contraction; ^6^ ACE: antegrade continence enema; ^7^ VP: ventriculoperitoneal; ^8^ ETV: endoscopic third ventriculostomy; * optimization declined.

## Data Availability

The original contributions presented in this study are included in the article/supplementary materials. Further inquiries can be directed to the corresponding author.

## Supplementary Materials

The following supporting information can be downloaded at: https://www.mdpi.com/article/10.3390/children12020184/s1, Figure S1: Facility clinical decision-making pathway; Figure S2: Facility treatment optimization and level of treatment pathway.
