# Supplementary material for: Colonic Manometry in Pediatric Patients with Spina Bifida: Results from a Retrospective Cohort Study
Source: Children (Basel). 2025 Feb 4;12(2):184. doi: 10.3390/children12020184 (PMC11854801; doi:10.3390/children12020184)
Supplement: Supplementary file 1 [file children-12-00184-s001.zip › children-3436086-supplementary.pdf]

**Supplemental Figure S1 Facility Clinical Decision-Making Pathway**

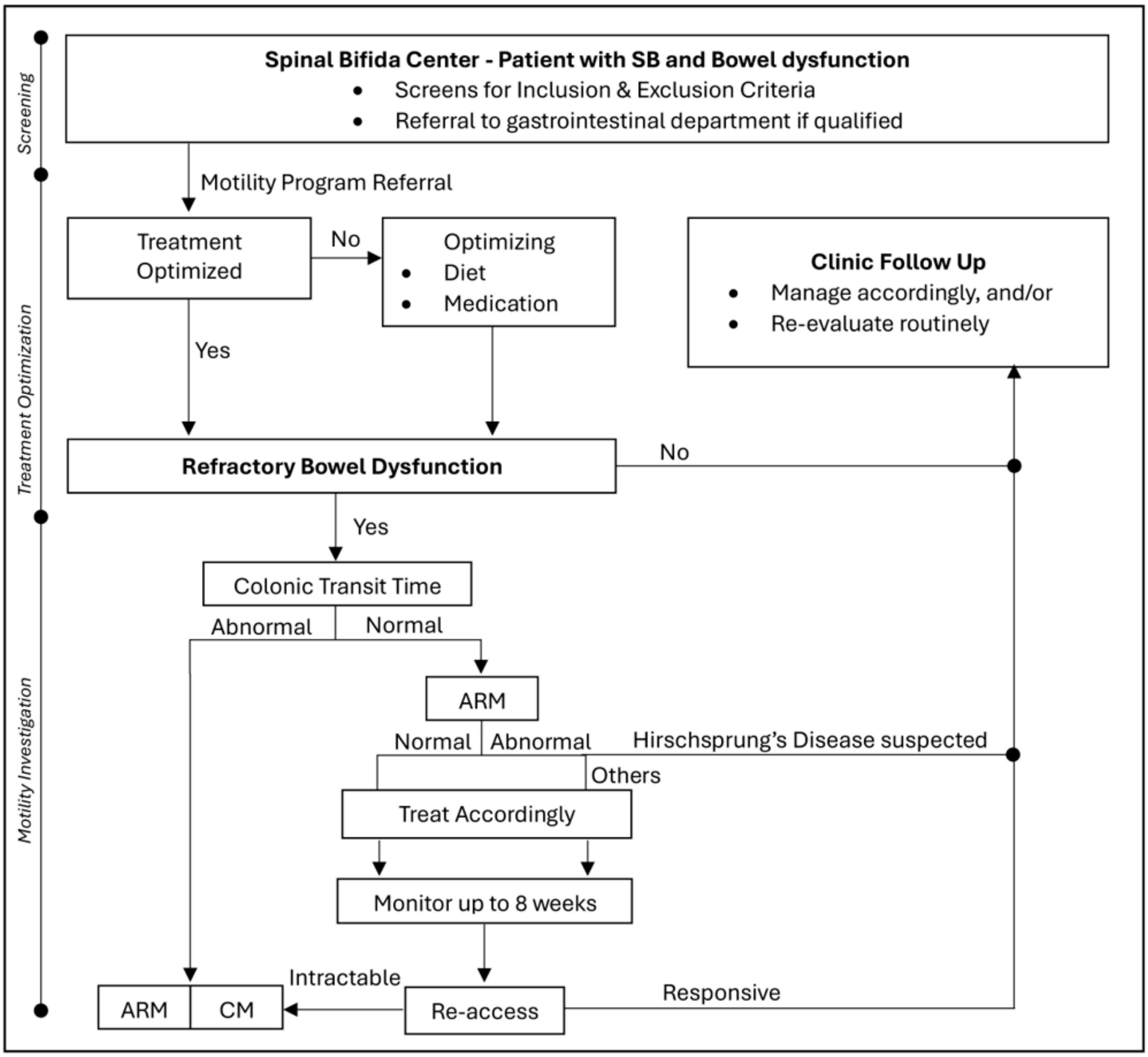

**Supplemental Figure S2 – Facility Treatment Optimization and Level of Treatment Pathway**

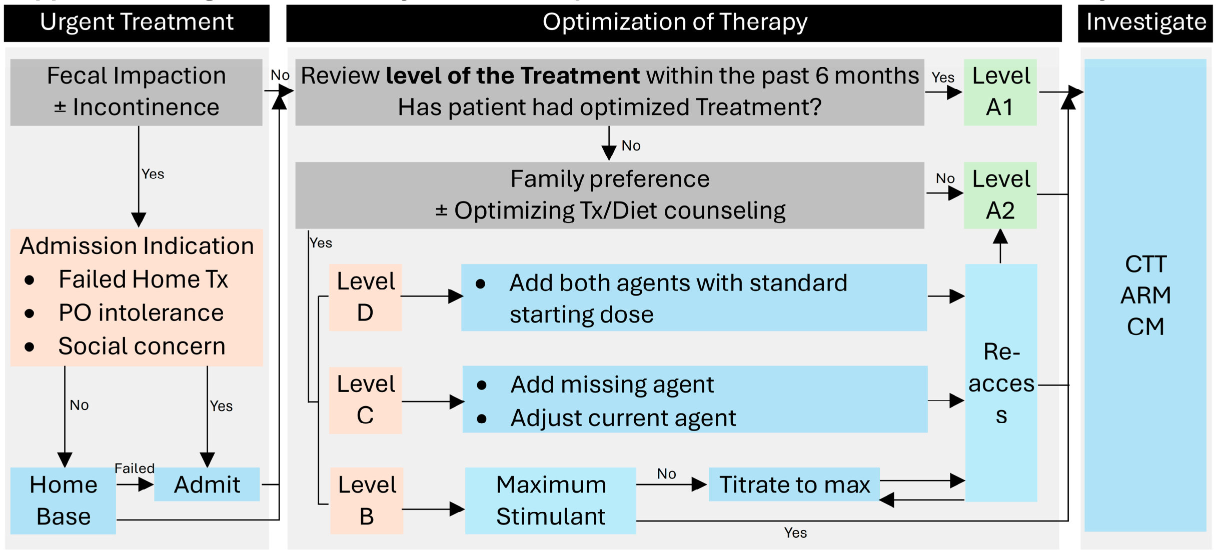


| Level | Condition (Enteral Medication) | Next Step |
|---|---|---|
| A1 | Optimal treatment achieved | → Arrange investigations |
| A2 | Not optimal, but family prefers to proceed with procedures | → Adjust the medication per pathway<br>→ Arrange investigations |
| B1 | On 1 stimulant<br><br>On osmotic agent | → Change to Bisacodyl; titrate Q1W until max (if applicable)<br>→ Otherwise, titrate Senna Q1W until max<br>→ Adjust osmotic agent accordingly |
| B2 | On 2 stimulants<br><br>On osmotic agent | → Titrate Bisacodyl Q1W until max (if applicable); stop 2nd agent<br>→ Otherwise, titrate Senna Q1W until max; stop 2nd agent<br>→ Adjust osmotic agent accordingly |
| C1 | On 1 stimulant<br><br>No osmotic | → Change to Bisacodyl; titrate Q1W until max (if applicable)<br>→ Otherwise, titrate Senna Q1W until max<br>→ Add osmotic standard dose, adjust accordingly |
| C2 | On 2 stimulants<br><br>No osmotic | → Titrate Bisacodyl Q1W until max (if applicable); stop 2nd agent<br>→ Otherwise, titrate Senna Q1W until max, stop 2nd agent<br>→ Add osmotic standard dose, adjust accordingly |
| C3 | No stimulant<br><br><br>On osmotic | → Start Bisacodyl (standard dose); titrate Q1W until max (if applicable)<br>→ Otherwise, start Senna (standard dose); titrate Q1W until max<br>→ Adjust osmotic agent accordingly |
| D | Missing both categories | → Start Bisacodyl (standard dose); titrate Q1W until max (if applicable)<br>→ Otherwise, start Senna (standard dose); titrate Q1W until max<br>→ Add osmotic standard dose, adjust accordingly |

Optimal treatment is defined as having intractable bowel dysfunction despite being on the maximum dose of oral/enteral stimulant combined with one or more other pharmacological therapies. The above outlines a stepwise approach for conventional maintenance therapy, aiming to achieve optimal treatment within 6 to 8 weeks, with additional details provided below:

- **Stimulant**: 1st line: Bisacodyl; 2nd line: Sennoside.
- **Osmotic**: 1st line: PEG 3350. If the maximum dose is reached, consider magnesium (if no contraindications). If PEG 3350 is not tolerated (e.g., volume issues), consider lactulose.
- **Rectal therapy** (manual disimpaction, suppository, enema): Rescue use only, indicated for overflow incontinence (encopresis, soiling), failure of oral/enteral cleanout, or pre-procedure preparation.
- Monitor stool texture during titration: If liquid fecal incontinence occurs, differentiate between full evacuation and smear soiling. For suspected impaction with overflow incontinence, increase the osmotic agent and consider rescue rectal therapy. If no impaction is suspected, evaluate for non-retentive fecal incontinence due to poor anal tone and decrease the osmotic agent.
